# Direct Measurement of B Lymphocyte Gene Expression Biomarkers in Peripheral Blood Transcriptomics Enables Early Prediction of Vaccine Seroconversion

**DOI:** 10.3390/genes12070971

**Published:** 2021-06-25

**Authors:** Dan Huang, Alex Y. N. Liu, Kwong-Sak Leung, Nelson L. S. Tang

**Affiliations:** 1 Cytomics Limited, Hong Kong Science and Technology Park, Hong Kong, China; cytomics.10@gmail.com (D.H.); cytomics03@gmail.com (A.Y.N.L.); ksleung@cse.cuhk.edu.hk (K.-S.L.); 2 Department of Computer Science and Engineering, The Chinese University of Hong Kong, Hong Kong, China; 3 Department of Chemical Pathology and Li Ka Shing Institute of Health Science, The Chinese University of Hong Kong, Hong Kong, China

**Keywords:** vaccination, seroconversion, B lymphocytes, gene expression, transcript abundance, influenza, COVID-19, biomarker, peripheral blood, transcriptome, PBMC

## Abstract

Peripheral blood transcriptome is a highly promising area for biomarker development. However, transcript abundances (TA) in these cell mixture samples are confounded by proportions of the component leukocyte subpopulations. This poses a challenge to clinical applications, as the cell of origin of any change in TA is not known without prior cell separation procedure. We developed a framework to develop a cell-type informative TA biomarkers which enable determination of TA of a single cell-type (B lymphocytes) directly in cell mixture samples of peripheral blood (e.g., peripheral blood mononuclear cells, PBMC) without the need for subpopulation separation. It is applicable to a panel of genes called B cell informative genes. Then a ratio of two B cell informative genes (a target gene and a stably expressed reference gene) obtained in PBMC was used as a new biomarker to represent the target gene expression in purified B lymphocytes. This approach, which eliminates the tedious procedure of cell separation and directly determines TA of a leukocyte subpopulation in peripheral blood samples, is called the Direct LS-TA method. This method is applied to gene expression datasets collected in influenza vaccination trials as early predictive biomarkers of seroconversion. By using TNFRSF17 or TXNDC5 as the target genes and TNFRSF13C or FCRLA as the reference genes, the Direct LS-TA B cell biomarkers were determined directly in the PBMC transcriptome data and were highly correlated with TA of the corresponding target genes in purified B lymphocytes. Vaccination responders had almost a 2-fold higher Direct LS-TA biomarker level of TNFRSF17 (log 2 SMD = 0.84, 95% CI = 0.47–1.21) on day 7 after vaccination. The sensitivity of these Direct LS-TA biomarkers in the prediction of seroconversion was greater than 0.7 and area-under curves (AUC) were over 0.8 in many datasets. In this paper, we report a straightforward approach to directly estimate B lymphocyte gene expression in PBMC, which could be used in a routine clinical setting. Moreover, the method enables the practice of precision medicine in the prediction of vaccination response. More importantly, seroconversion could now be predicted as early as day 7. As the acquired immunology pathway is common to vaccination against influenza and COVID-19, these biomarkers could also be useful to predict seroconversion for the new COVID-19 vaccines.

## 1. Introduction

Vaccination by controlled exposure to an antigen or its precursor is a good strategy for prevention of full-blown infection. Such prior exposure activates the acquired immune system to produce antibodies against the pathogen. In vaccinated individuals, the pathogens will be controlled quickly, and symptoms of infection are alleviated. A common example is vaccination against the influenza virus. As the prevalent strains of influenza virus change frequently, annual vaccination of different influenza virus strains is a common practice. Of particular relevance is the current pandemic COVID-19 caused by severe acute respiratory syndrome coronavirus 2 (SARS-CoV-2), against which a worldwide vaccination campaign is also underway.

In the case of influenza vaccination, only about half of those vaccinated individuals mount substantial immune responses against the antigens, which is known as seroconversion. They are called responders (R) to vaccination and the remaining responses are called non-responders (NR). However, antibody production, measured in the unit of titers representing the level of antibodies against the vaccination antigen in blood samples, can only be known 28 days after vaccination [1]. In order to practice precision medicine in a vaccination program, an early response biomarker that can predict seroconversion much earlier is desirable. This is useful so NR could be identified early and other protection measures could be implemented.

Blood transcriptome biomarkers is a subject undergoing intensive research [2,3,4]. In the application to vaccination research, gene expression changes in blood samples were studied in various vaccination trials towards different pathogens, including influenza, tuberculosis, hepatitis, and yellow fever [5,6,7,8]. These studies measured gene expressions in peripheral blood samples (examples include Whole blood, WB and PBMC) containing a mixture of various leukocyte subpopulations. A few of them also quantified transcript abundance (TA, a technical term of quantification for gene expression) of leukocyte subpopulations for selected leukocyte cell-types after cell sorting [6]. Systemic biology approaches were applied to study the complexity problem of molecular signatures after vaccination, which was largely confounded by the presence of various leukocyte subpopulations in clinical samples, as reviewed by Pezeshki et al. [9]. Previous studies of TA after vaccination were limited to either focusing on the genetic determinants of responders or the difference between vaccination and a full-blown infection [10].

Most vaccination transcriptome studies used PBMC samples, while only a few studies used WB samples [11]. These two kinds of blood samples are both cell mixture samples composed of various leukocyte subpopulations or cell-types, such as neutrophils, lymphocytes, monocytes, etc. So, the results are convoluted and confounded by different proportional cell counts of each of the cell subpopulations in these cell mixture samples. The research community has called for new biomarkers to enhance vaccine development, particularly in view of the current COVID-19 pandemic [9,10,11,12]. Here, we are interested to know if B lymphocyte TA could be a reliable early predictor for subsequent seroconversion of B lymphocyte as the precursor of plasma cells. B lymphocyte is the production warehouse of antibodies, making it the most logical choice of cell-type to study. In order to obtain TA levels of a single subpopulation (cell-type) in peripheral blood, prior isolation and purification of the specified cell-types are required under the conventional approach. However, they can only be carried out in a research laboratory setting, as the cell isolation procedures are too tedious to be used in a routine hospital laboratory. Recently, single-cell RNA-sequencing (scRNA-seq) has also been used to obtain gene expression information of every individual cell in a cell mixture, which has been applied to obtain the B cell immunoglobulin gene repertoire data [13]. Although single leukocyte subpopulation TA can be obtained by a cell separation procedure, single cell digital PCR or scRNA-seq, these methods are either too labor intensive or expensive precluding a readily translational use in routine healthcare settings.

To improve our understanding of the cellular origin of a particular gene transcript commonly used in peripheral blood mixture samples, a workflow flowchart was derived to define a list of cell-type informative genes in a peripheral blood mixture sample (Supplementary Document: flowchart). In essence, the framework first shortlists cell-type informative genes that have the majority (>50%) of their mRNA transcripts having originated from the specified cell-type (B lymphocyte in this study) inside a given cell mixture sample (e.g., PBMC or WB). These lists are defined for a given cell mixture sample with a respective typical proportional cell count of various component cell-types. A new biomarker called Direct B lymphocyte leukocyte subpopulation transcript abundance (Direct LS-TA) derived from the ratio of TAs of two informative genes (including a B lymphocytes target gene and a B lymphocyte reference gene) can be directly quantified from peripheral blood samples without the need to purify B lymphocytes.

A mathematically based methodology (known as deconvolution) has been used to determine the proportional cell counts of various subpopulations in cell mixtures, which requires a long list of signature genes for each subpopulation [14,15,16]. However, the primary motivation of deconvolution is fundamentally different from our method. Our primary objective is to determine TA of genes of a leukocyte subpopulation of interest, whereas the proportional cell count of a subpopulation was the primary target in deconvolution.

With the list of B lymphocyte target genes identified with our model, we could estimate their expression in the B lymphocytes directly in peripheral blood. We analyzed these B lymphocyte TA biomarkers in publicly available datasets of vaccination trials. As we were interested in identifying early B lymphocyte TA biomarkers that can predict subsequent seroconversion, we explored the predictive performance of the new biomarkers.

## 2. Materials and Methods

### 2.1. Datasets Used in the Analysis of Gene Expression of Peripheral Blood and B Cells

In order to identify B lymphocyte cell-type informative genes that can predict vaccination response, the following datasets of gene expression obtained from peripheral blood samples were used (Table 1). These datasets were available in the Gene Expression Omnibus (GEO) maintained by the United States National Institute of Health. The details can be obtained under their accession number. The most common type of blood sample was PBMC. In some datasets (e.g., GSE45764), further purification of specific cell-types or leukocyte subpopulations was performed, such as by obtaining TA of the purified B lymphocytes [6]. There are other additional datasets of vaccination studies in GEO. However, only these datasets had participants’ antibody response data, HI titer, which allowed us to define responders and non-responders. GSE29615 only obtained one responder by our definition and was not further analyzed.

### 2.2. Definitions of Vaccination Response

Responders (R) after vaccination were defined following the criteria of a seroconversion/significant increase of anti-hemagglutinin antibody levels (HI titer) performed by hemagglutination inhibition (HI) assays on subjects’ plasma or serum which was taken before and after vaccination (commonly taken on Day 28) [1]. The European Committee for Medicinal Products for Human Use (CHMP) defines a seroconversion/significant increase among responders as: (a) HI titer after vaccination is at least 1 in 40 and (b) at least a four folds increase from the pre-vaccination baseline [1,19]. Individuals who did not meet these criteria after vaccination were defined as non-responders (NR).

### 2.3. To define B Lymphocyte Informative Genes Whose Expression Level Can Be Reliably Inferred in Cell-Mixture Samples, e.g., PBMC or WB

An overview of the workflow to define B lymphocyte informative genes and their application in the meta-analysis is shown in a flowchart in the Supplementary Workflow Document File.

**Figure 1:**
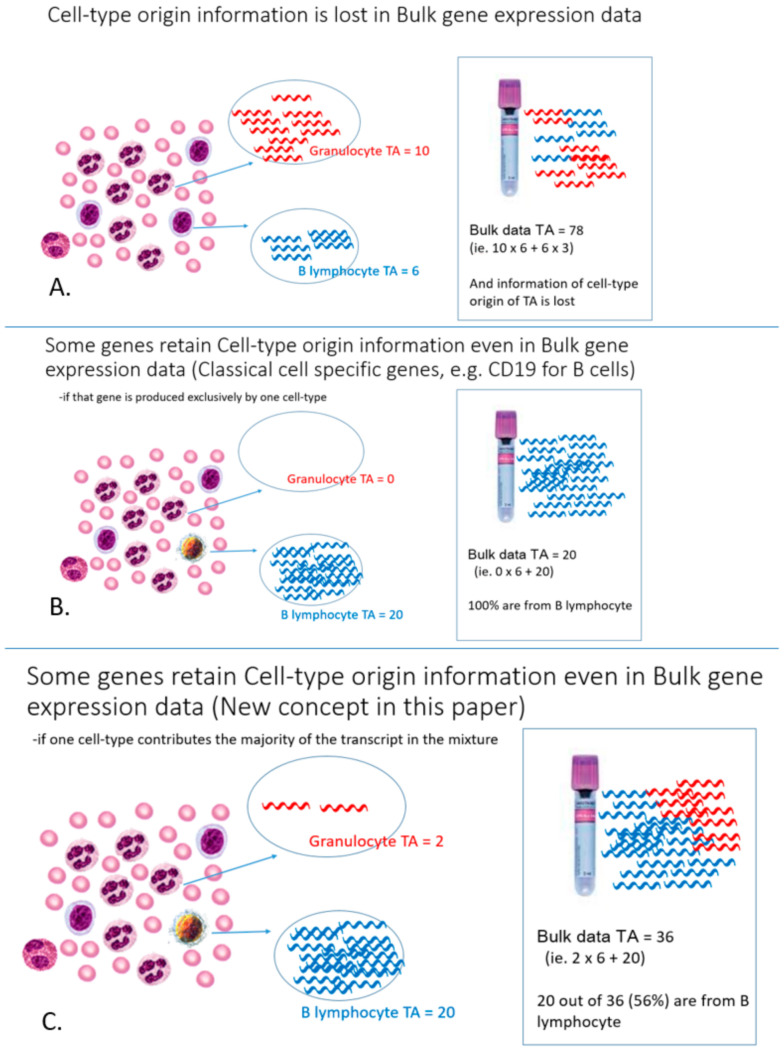
(**A**) Cell-type origin information is lost in bulk gene expression data. (**B**) The conventional concept of cell-type-specific genes are those genes exclusively produced by one particular cell-type. Such genes would be few in number. (**C**) Our new concept: Cell-type informative genes are predominantly expressed by a single cell-type, which is the sole major contributor (>50%) of gene transcripts in a cell-mixture sample (e.g., B lymphocyte in the figure). Transcript symbols produced by various cell-types are colored for presentation purposes only (Red for granulocyte produced transcripts and blue for B-lymphocyte produced transcripts); the transcripts are, in fact, identical.

**Figure 2:**
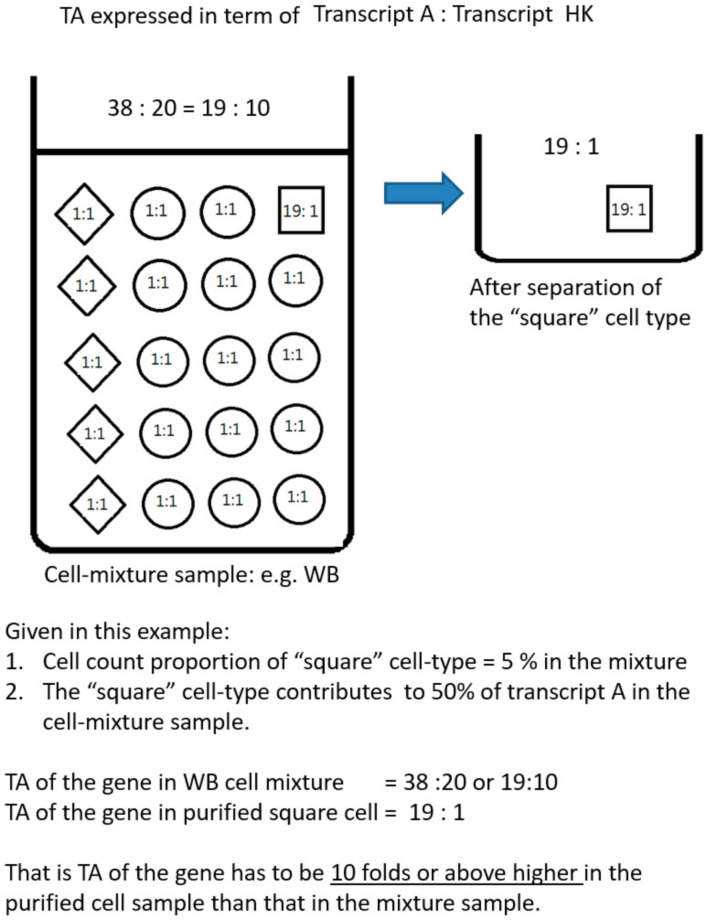
A theoretical model of defining B lymphocyte (represented by square symbols) informative genes in a WB sample (with three different cell-types, symbolized as square, circle, and rhomboid). A pre-defined proportional cell count of 5% is given as the proportion of B lymphocyte in WB. When TA of a gene was measured both in the WB and purified B lymphocyte samples, B lymphocyte informative genes as we defined them would have at least a 10-fold higher expression in the purified cell sample (purified B cells) than the cell-mixture sample (WB). Measurement of TA is based on a commonly used normalization approach by using a conventional housekeeping (HK) gene. (see also reference [22]).

Figure 1 depicts the problem and explains the difference between the new concept cell-type informative genes and classical cell-type specific genes. In our approach, cell-type informative genes are predominantly expressed by only a single cell-type (e.g., B lymphocytes) to the extent that that cell-type is the sole major contributor (>50%) of gene transcripts in the cell-mixture sample. Intuitively, these genes must have a higher expression in B lymphocyte than the cell mixture sample by a certain threshold before B lymphocytes could be the sole major contributor. We found that this threshold was related to the proportional cell count of the cell-type of interest. In other words, we can empirically derive this threshold value from the proportional cell count (Figure 2 and Supplementary Figure S1). For the example of B lymphocytes in WB, such an informative gene must have a much higher expression in B lymphocytes compared to other cell types so that ~5% population of B lymphocytes could be the sole major producer of the gene transcripts in WB. These genes are called cell-type informative genes, in order to contrast them with the conventional concept of cell-type specific genes, which are exclusively produced by a particular cell-type.

To identify B lymphocyte informative genes among over 10,000 genes that are expressed in hematological cells for downstream analysis, a mathematic model was used to shortlist putative genes by incorporating an expected proportional cell count of a particular cell-type (B-lymphocyte in this study) in a cell-mixture sample (e.g., PBMC or WB). Although the B lymphocyte count is variable among individuals and decreases with age [20,21], a ballpark figure for the proportional cell percentage of B-lymphocyte in WB of 5% was obtained, which was sufficient for the model to work [20]. Similarly, B lymphocytes were expected to account for ~10% of the cells in PBMC.

The model is explained in more detail in Figure 2, which describes a scenario in which a cell-type accounts for 5% in a cell mixture (similar to the situation of B lymphocytes in WB). Gene expression (TA) was measured, for example together with a standard housekeeping gene by some methods such as qPCR, microarray or RNA-seq. TA was measured in both the cell mixture and the purified component cell samples. It shows that any genes whose expressions are 10-folds higher in the purified cell sample than the cell mixture would qualify to be potential cell-type informative genes, as the component cell would be possibly the sole major contributor.

As transcripts of these informative genes predominantly come from a single cell-type, direct measurement of their total TA in the cell mixture sample would provide a valid estimation of the gene expression level of that component cell-type. However, we needed a reference gene for normalization for the biological variation in the proportional cell counts among individual samples. Instead of using traditional housekeeping genes (like *GAPDH*, *UBC*), one (or more) reference gene was chosen among the informative genes with an additional requirement of having a low biological variation. This was because conventional housekeeping genes are produced by all the different cell-types in the cell mixture, so it only represents the total cell count of all those various cell-types present in the cell mixture sample but does not represent the cell count of B lymphocytes. Therefore, the normalization factor also must have been a cell-type informative gene. The biological variation could be calculated as inter-individual variance in the purified cell-type samples and expressed as a coefficient of variation (CV%).

In this study, FCRLA was chosen as a reference gene, the Direct LS-TA biomarker was expressed as the ratio of TA of a B lymphocyte informative gene to TA of FCRLA. The performance of Direct LS-TA could be assessed by the correlation between the Direct LS-TA gene biomarker (TA target gene/TA FCRLA in PBMC) and its ground-truth, TA of that target gene in purified B lymphocytes (TA target gene/TA standard housekeeping gene in purified B lymphocyte samples). The Direct LS-TA method can be applied to more than 10 target genes and B lymphocyte expression of these genes could be reliably determined directly in PBMC.

### 2.4. A Mathematical Model to Generalise the Informative Gene Framework and to Define the Fold Difference Threshold (Supplementary Figure S1)

A mathematical model to identify subpopulation informative genes was derived to determine the required fold difference threshold between a purified cell sample and a cell mixture sample for any given proportional cell count. The threshold value facilitates the identification of cell-type informative genes for various scenarios of different cell-types in different cell-mixture samples (e.g., WB, PBMC or another tissue) [22].

### 2.5. Model of % Contribution of the Transcript by a Cell Type (e.g., B Cells) inside a Cell Mixture (e.g., WB)

The following model can be used to determine the relationship between % contribution by a cell type based on the differential gene expression of purified cell samples and a cell-mixture sample.

(1) $$TA_{WB}=P_{B\;cells}\cdot TA_{B\;cells}+\left(1-P_{B\;cells}\right)\cdot TA_{other\;cells}$$

Let
$TA_{WB}$
= total transcript abundance (TA) of a gene in a WB sample

$TA_{B\;cells}$
= TA of a gene in B cells present inside the WB sample

$P_{B\;cells}$
= proportional cell count of B cells inside the WB sample (e.g., 5%) This value is assigned based on general knowledge or (in other settings) could be estimated from sample data by methods like deconvolution using TA data of other genes.

$TA_{other\;cells}$
= transcript abundance of all other cell-types based on their respective proportional counts. (This is a hypothetical parameter that is not actually determined or measured but is used to present a general model.)
X= fold change difference between $TA_{B\;cells}$ and $TA_{WB}$, = $\frac{TA_{B\;cells}}{TA_{WB}}$ This is the observed difference in expression of a gene purified B cell samples and WB.

In order to understand the relationship between % contribution of TA in a cell mixture (WB) by a specified cell-type (B cells, *Y*-axis in Supplementary Figure S1) and the threshold fold difference (*X*-axis).

% TA contribution in a cell-mixture sample by B cells

(2) $$\frac{P_{B\;cells}\;TA_{B\;cells}}{TA_{WB}}=\frac{P_{B\;cells}\;TA_{B\;cells}}{\frac{TA_{B\;cells}}{X}}\;=P_{B\;cells}\;X.$$

The expression indicates that the relationship between % *TA* contribution and fold-change difference (*X*) between specified cell type and *WB* is linear with the slope defined by the proportional cell count of that cell type.

Supplementary Figure S1 shows such a relationship for the two scenarios: (1) B cells in WB (given proportional cell count as 5%); and (2) B cells in PBMC (given proportional cell count as 10%). Genes with 10-fold or higher TA in purified B cells than WB would have B cells contributing at least 50% of transcripts in the WB samples. Similarly, genes with 5-fold or higher TA in purified B cells than PBMC are B lymphocyte informative genes in PBMC.

### 2.6. Identifying B Lymphocyte Informative Target and Reference Genes in PBMC Using Real Datasets

A RNA-seq dataset (GSE45764) had transcriptome data of both purified B lymphocytes and PBMC. Therefore, the datasets were used to identify B lymphocyte informative genes. Next, biological variation was determined for all B lymphocyte informative genes and expressed in terms of CV%. TNFRSF13C and FCRLA were selected as a B lymphocyte reference genes, respectively, which were used as the denominator in the new Direct LS-TA biomarker parameter to infer B lymphocyte expression of the target genes.

As log transformation had been applied to the TA data, this new biomarker parameter was calculated as the difference (subtraction) between the B lymphocyte informative target gene and the B lymphocyte informative reference gene. Using TNFRSF17 as the target gene, for example, its Direct LS-TA could be expressed as:

Direct B lymphocyte LS-TA of TNFRSF17 = log(TNFRSF17) − log(TNFRSF13C).

**Figure 3:**
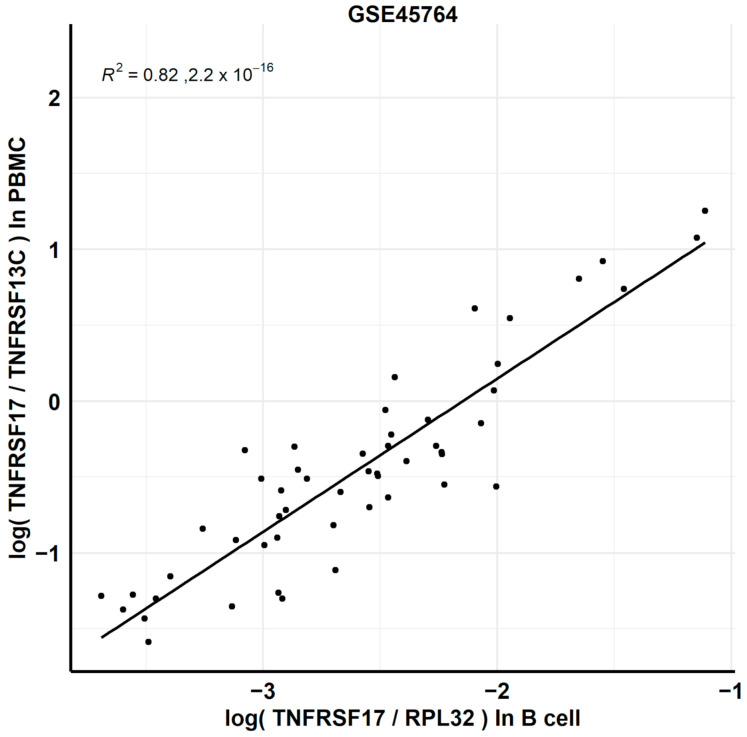
High level of correlation between new biomarker, Direct LS-TA biomarker of target gene TNFRSF17 by obtaining a ratio of TAs of two genes (TNFRSF17 and TNFRSF13C) in PBMC samples (*Y*-axis) and TA of TNFRSF17 in purified B lymphocytes (ground-truth, *X*-axis). R^2^ = 0.82 (*p*-value < 2.2 × 10^−16^).

As the dataset GSE45764 provided RNAseq transcriptome data of paired PBMC and purified B lymphocytes samples taken at the same time. The ability of the Direct B lymphocyte LS-TA resulted in reflecting the ground-truth, while TA of the same target gene in purified B was evaluated by the correlation between the Direct B lymphocyte LS-TA in the PBMC samples and the TA of the target genes in the purified B lymphocytes. Pearson’s correlation coefficient (r) was used. A conventional housekeeping gene (e.g., RPL32) was used to normalize the TA results in the purified B cell samples (Figure 3).

Using this exploratory dataset, the Pearson’s correlation coefficient (r) values of all shortlisted B lymphocyte informative genes were determined, and only those with r > 0.8 were further analyzed for their differential expression between R and NR groups.

In addition, several other datasets (GSE59635, GSE59654, GSE59743) also contained microarray expression data of both PMBC and purified lymphocytes samples. They were also used to validate the list of B lymphocyte informative genes, reference genes and the performance of Direct B lymphocyte LS-TA of the selected target genes.

### 2.7. Meta-Analysis of Differentially Expressed B Lymphocytes LS-TA Genes in Other Vaccination Datasets

To have a better idea of the reproducibility of these findings, meta-analyses were performed for these four Direct B lymphocyte LS-TA biomarkers with PBMC data in a collection of datasets that were performed on different platforms (including Affymetrix and Illumina microarrays).

The datasets were downloaded from GEO. Direct LS-TA results were determined from expression intensities of the target genes and reference genes that had been picked up in the discovery dataset. Fixed effect meta-analysis was performed using R package meta.

### 2.8. Datasets Quality Control and Statistic Analysis

The Microarray datasets were checked to see whether they had been normalized by RMA normalization or quantile normalization. All data were also log-transformed with base 2. The quality check of the datasets included a check for outliners by Mahalanobis distance metrics [23,24] using: (1) a list of common housekeeping genes; and (2) a list of recognized cell-type-specific genes. Samples in a dataset were defined as outliners and removed if they both failed the outliner tests in Mahalanobis distance metrics of (1) and (2). An example of outliner identification in Dataset GSE59654 is shown in Supplementary Figure S2.

Statistics analyses were performed with R packages, including plotROC, meta and stats.

## 3. Results

### 3.1. B Lymphocyte Informative Genes and Reference Genes

A list of B lymphocyte informative genes is shown in Table 2, together with their biological CV% in the purified B cell samples. Genes with the least CV% include well-recognized B lymphocyte markers, like CD19, CD20 (MS4A1) and CD22. CV% of these genes range from 15% to 18%. Interestingly, TNFRSF13C and FCRLA have similar CV% values to these well recognized B cell-specific markers. Therefore, they were further explored as B lymphocyte reference genes and used as the denominator for the new Direct LS-TA biomarker to predict vaccination response.

### 3.2. Using Direct B lymphocyte LS-TA Biomarker in PBMC to Infer Expression (TA) of Target Gene in Purified B lymphocytes

The correlation between the new Direct LS-TA biomarkers in PBMC samples and the ground-truth TA in purified B lymphocytes was used to assess its ability to infer expression of the target genes in purified B lymphocytes.

Figure 3 and supplement Figure S3 show the performance of the Direct B lymphocyte LS-TA of TNFRSF17 measured in PBMC as a biomarker reflecting TNFRSF17 gene expression in purified B lymphocytes. Direct LS-TA of TNFRSF17 is shown on the *Y*-axis with the expression level of that gene in paired purified B lymphocytes shown on the *X*-axis. The strong correlation (R2 = 0.82, *p* < 2.2 × 10^−16^) results confirmed that Direct LS-TA TNFRSF17 in PBMC is a reliable indicator of TNFRSF17 expression in B lymphocytes.

Similarly, Direct LS-TA for another target gene (TXNDC5) in PBMC was also representative of its expression in B lymphocytes. This was shown in both RNAseq data and microarray validation data. With such a strong correlation, Direct LS-TA quantification was applied to other vaccination response datasets in which only PBMC samples were collected. In fact, the great majority of vaccination response datasets did not have expression information for purified leukocyte subpopulations. The feasibility of using the Direct LS-TA method provides a means to analyze cell-type specific TA in these and many other cells-mixture datasets.

Not only could the B-lymphocyte expression of TNFRSF17 be inferred directly in a peripheral blood sample, but a list of other B-lymphocyte informative genes (Table 3, using TNFRSF13C as reference gene) could also be analyzed using the Direct LS-TA method. The Direct LS-TA biomarker of these target genes had a very good correlation with their TA in purified B lymphocytes.

Many target genes encode important proteins for antibody production (like *IGHG1* and *IGLL5*). Other target genes include *MZB1*, *FCRL5*, *TNFRSF17* and *TXNDC5*, which have been associated with various essential functions of B lymphocytes.

### 3.3. Application of Direct LS-TA in Vaccine Response PBMC Transcriptome Data

#### 3.3.1. Differential Expression of Direct B Lymphocyte LS-TA in the First Week after Vaccination

**Figure 4:**
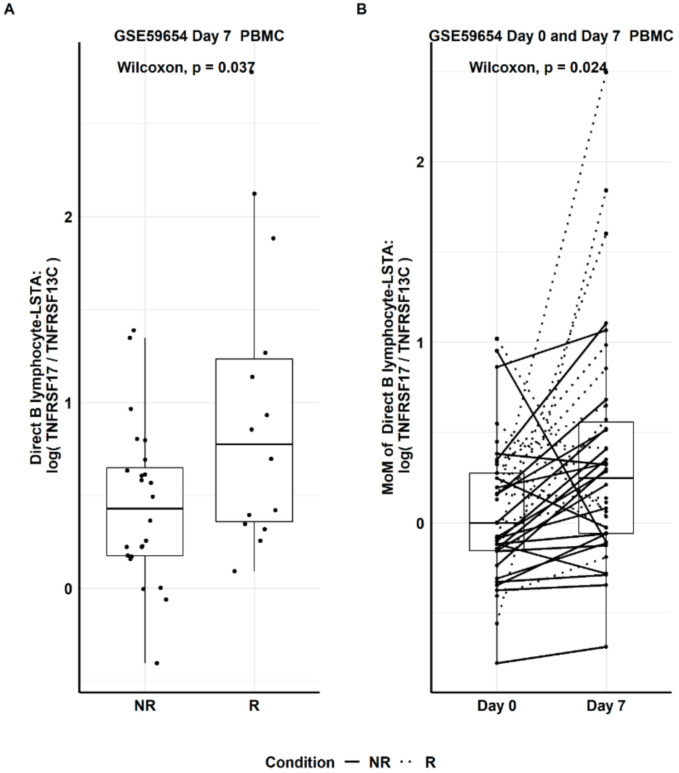
Based on the explorative dataset, GSE59654, (**A**) Day 7 Direct B-lymphocyte LS-TA results of TNFRSF17 in responder (R) and non-responder (NR) groups. (**B**) Paired Day 0 and Day 7 change in Direct LS-TA expressed as multiples of median (MoM) using the Day 0 group median.

With this new B lymphocyte biomarker available (Table 4), it is now possible to estimate the expression of B lymphocyte as a response to vaccination using the other expression datasets with only PBMC samples available (see the list in Table 1). Dataset GSE59654 (influenza vaccine) was used as an exploratory dataset to detect any differential gene expression (as determined by Direct LS-TA) on Day 7 between the two groups (i.e., R and NR groups). This dataset was selected because it had the largest sample size. Figure 4A shows that R had a significantly higher level of Direct B lymphocyte LS-TA of TNFRSF17 than that of NR (Wilcoxon test, p-value = 0.037). Similarly, the increment of Direct B lymphocyte LS-TA of TNFRSF17 from the day of vaccination (Day 0) to Day 7 was also significant in a paired Wilcoxon test (Figure 4B, *p*-value = 0.024).

**Figure 5:**
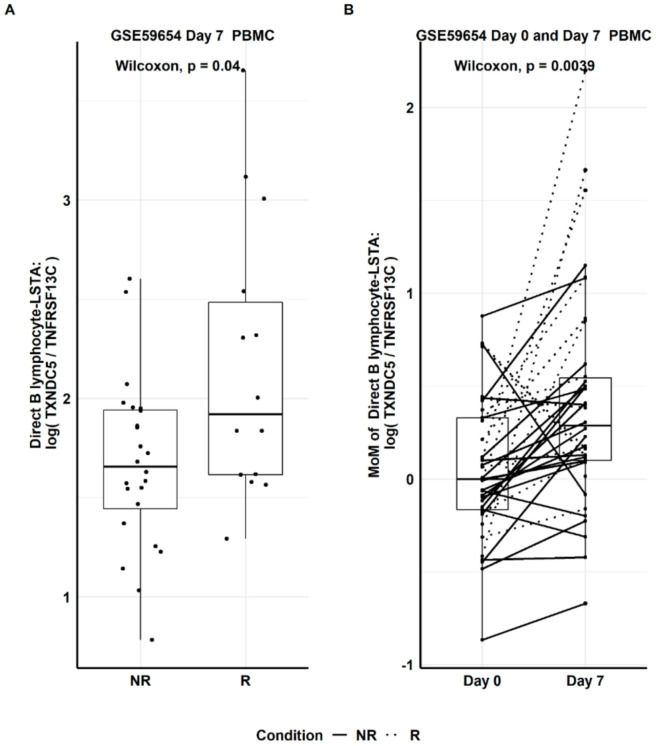
(**A**) Day 7 Direct B-lymphocyte LS-TA results of another target gene TXNDC5 in responder (R) and non-responder (NR) groups. (**B**) Paired Day 0 and Day 7 change in Direct LS-TA expressed as multiples of median (MoM) using the Day 0 group median.

Similarly, Direct LS-TA of another target gene TXNDC5 (both Direct B lymphocyte LS-TA of TXNDC5 on Day 7 and the increments from Day 0 to Day 7) were also significantly different between responders and non-responders (Figure 5).

#### 3.3.2. Meta-Analysis of Differentially Expressed B lymphocytes LS-TA Genes in Other Datasets

To have a better idea of the reproducibility or replication of these findings, meta-analyses were performed for these four Direct B lymphocyte LS-TA biomarkers in PBMC data in a collection of datasets. These data were obtained from different transcriptome quantification platforms (Affymetrix and Illumina microarrays).

**Figure 6:**
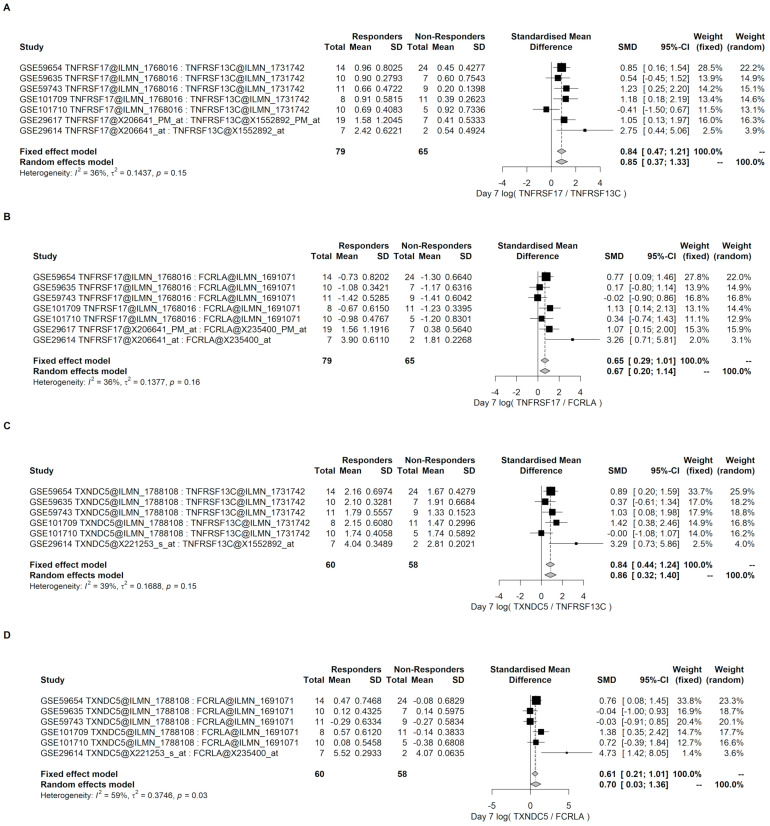
Meta-analysis of the Day 7 results of Direct B lymphocyte-LSTA of TNFRSF17 and TXNDC5 using TNFRSF13C or FCRLA as a reference gene in PBMC datasets. Day 7 Direct B lymphocyte-LSTA was significantly higher among the responder groups with an overall effect SMD of 0.6 to 0.8. The specific probe sets used in the calculation of Direct LS-TA are also shown in the table following the gene symbols. (**A**,**B**) are the results of Direct LS-TA of TNFRSF17 using two different B lymphocyte reference genes (TNFRSF13C and FCRLA). (**C**,**D**) show the Direct LS-TA results of TXNDC5. The specific probe sets used in the calculation of Direct LS-TA are also shown in the table following the gene symbols.

The meta-analysis on TNFRSF17 was performed on 7 PBMC datasets (Table 1 and Figure 6). Regardless of whether TNFRSF13C or FCRLA was the reference gene, the meta-analysis confirmed an overall differential level of Direct LS-TA TNFRSF17 on Day 7 between NR and R groups. In fact, the overall effects were similar in magnitude, and their SMD ranged from 0.6 and 0.8. When TXNDC5 was used as the target gene, similar results were obtained. This suggests that either TNFRSF17 or TXNDC5 and their respective Direct B lymphocyte LS-TA could be used as an early biomarker for vaccination response.

**Figure 7:**
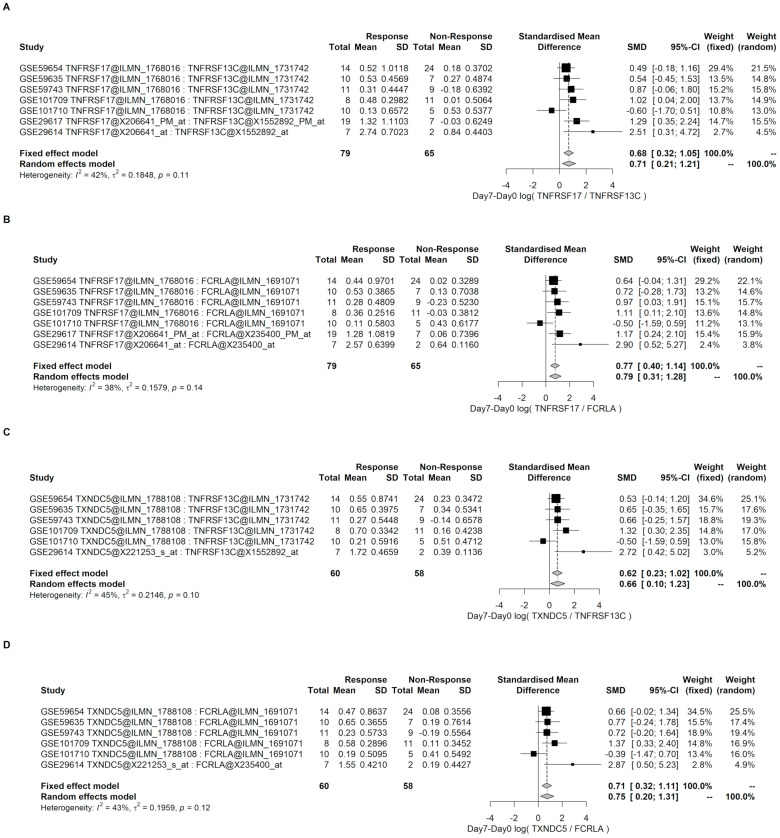
Meta-analysis of the differential increment from Day 0 to Day 7 of Direct B lymphocyte-LSTA between R and NR groups. (**A**,**B**) are the results of the differential increment of Direct LS-TA of TNFRSF17 using two different B lymphocyte reference genes (TNFRSF13C and FCRLA). (**C**,**D**) are the results of the differential increment of the Direct LS-TA results of TXNDC5. Direct B lymphocyte-LSTA showed significantly higher increments among the responders with an overall effect SMD of between 0.62 to 0.77. The specific probe sets used in the calculation of Direct LS-TA are also shown in the table following the gene symbols.

Similarly, the increment from Day 0 to Day 7 was analyzed by meta-analysis in these datasets. The overall effects of greater increment in the responders were confirmed (Figure 7).

### 3.4. Evaluation of the Performance by Receiver Operator Curve (ROC) Analysis

**Figure 8:**
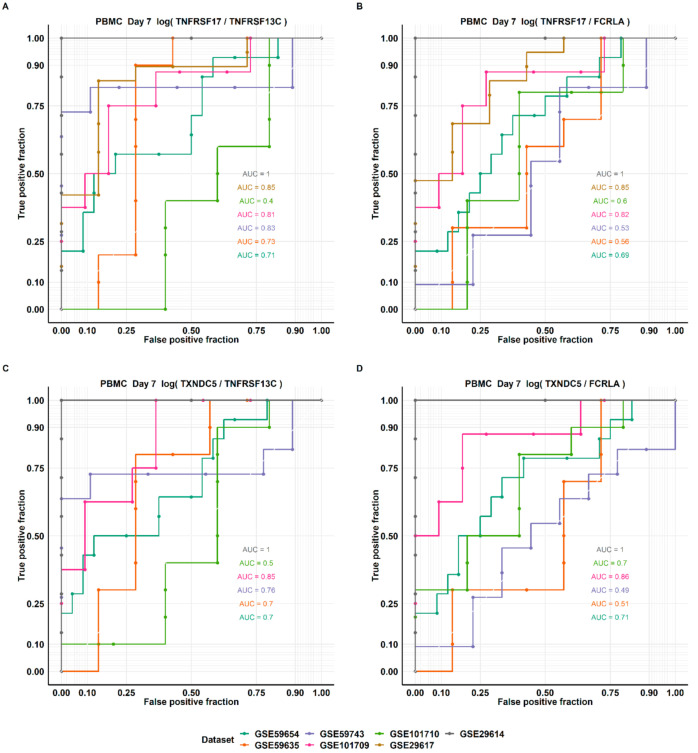
ROC curves of Day 7 Direct B lymphocyte LS-TA of TNFRSF17 (**A**,**B**) and TXNDC5 (**C**,**D**) using two different B lymphocyte informative reference genes (TNFRSF13C and FCRLA). The performance of the single cell-type biomarker parameters was analyzed by ROC with responder as a positive outcome.

Receiver operator curve (ROC) analysis was used to evaluate the test performance of Direct LS-TA as an early (first-week) predictive biomarker for subsequent seroconversion on Day 28. As the case-mixes were different among studies, the Area Under Curve (AUC) values were also different (Figure 7). All studies had AUC values greater than 0.5, and a few studies’ AOC values were higher than 0.8. In some settings, the sensitivity could be 0.8 or higher, while the specificity was around 0.5. On the other hand, a high specificity of the range of 0.8-0.9 could be targeted if a lower sensitivity down to 0.6 is allowed. Figure 8 shows the ROC analysis of the increments of Direct B lymphocyte LS-TA from Day 0 to Day 7 as biomarkers. Again, most studies have AUC values above 0.6.

The exact cut-off point to be used depends on the purpose and rationale of the test. The applications of this method are wide. For example, if the vaccine is unstable, the producer would want to know early if any batch of the vaccine had degraded; this test would provide the answer as early as seven days after administration of the batch. In this scenario, a group of vaccine recipients will be monitored for the Day-7-increase in Direct B lymphocyte LS-TA. A high sensitivity cut-off value would be used, so most of the recipients are expected to reach the cut-off value indicating that the vaccine is active. If the number or percentage of recipients showing an increase in Direct B lymphocyte LS-TA on Day 7 is less than a pre-defined percentage, degradation of the vaccine batch would be possible and remedial work could be implemented early rather than waiting for the seroconversion data after Day 28.

In addition, the Direct B-lymphocyte LS-TA test could be used for personalized vaccination in which an individual wants to know his subsequent vaccination response early after vaccination. A high specificity cut-off value would be used in this scenario. Although only around 60% of the responder recipients would achieve this cut-off value, the false position rate is low. This supports a high positive predictive value, so recipients with positive Direct B-lymphocyte LS-TA results would be very likely to be responders. The consequence of a negative Direct B-lymphocyte LS-TA is a requirement of doing a follow-up antibody test on Day 28. The high positive rate of Day 7 Direct B lymphocyte LS-TA allows the majority of responders to obtain their vaccine response results early.

## 4. Discussions

The Direct LS-TA biomarker is a simple B-lymphocyte informative TA biomarker that allows direct determination of B lymphocyte gene expression in PBMC samples without the need to purify B lymphocytes from other blood cells.

This approach is different from deconvolution methods of deriving the proportional cell counts of various cell-type subpopulations [17]. On the other hand, our method is focused on direct estimation of the average gene expression (TA) of target informative genes of a given subpopulation in WB or PBMC. Furthermore, this Direct LS-TA method also has a key advantage that it is highly versatile, as it can be applied to TA data obtained from various quantification platforms.

Previous studies including those datasets we analyzed here provided comprehensive and long lists of differential expression genes (DEGs) typically composed of tens or hundreds of genes [6]. Then, pathway or gene set enrichment analysis was used to attribute them to various pathways (such as interferon or other cytokine response) [7,17,18]. However, such a long list of DEGs is not very helpful to derive a simple biomarker that can be used in the clinic. Other groups proposed a modular approach to group DEGs into groups according to their pathway or function [3]. There are 382 modules while each has a dozen of genes, and software was used to interpret change of TA inside modules [25]. Nonetheless, interpretation of fingerprint profile output from these 382 modules is not easy. Furthermore, the confounding factor of variation of proportional cell counts of various leukocyte subpopulations is not accounted for.

On the other hand, the new biomarker using a ratio of TA of only two cell-type informative genes is both technically straightforward and easy to interpret. Here, the key hurdle is to identify these cell-type informative genes for which their TA could be readily estimated in cell mixture samples. In this paper, we showed a panel of genes that could be used for this purpose and their application in a real life situation.

Based on a similar concept as our cell-type informative genes, the latest human protein atlas [26] also has similar gene lists, which are called lineage enriched genes. For example, there are 50 such B cell lineage enriched genes with the highest expression in the blood (https://www.proteinatlas.org/search/blood_cell_lineage_category_rna%3Ab-cells%3BLineage+enriched+AND+tissue_category_rna%3ABlood%2CLymphoid+tissue%3BIs+highest+expressed+AND+sort_by%3Atissue+specific+score (accessed on 4 May 2021)). Here, the two terms, B lymphocytes informative genes and B lymphocyte lineage enriched genes are conceptually related but the gene lists are slightly different, as the methods of calculation are different. For example, key target genes (e.g., TNFRSF17) used as the biomarker in this paper are not included in the protein atlas cell-type enriched gene list of B lymphocytes. Here, we chose two reference genes (TNFRSF13C and FCRLA), as they had low CV% similar to other known B lymphocyte-specific genes (e.g., CD19, CD22). Other reference genes could also be used but prior validation was required.

### Limitations of the Study

Our analysis was limited by (1) availability of study datasets and (2) having little control of the study protocol of the original study. For example, there were studies using different gene expression quantification platforms, ranging from microarrays to RNA-sequencing. Anyway, the versatility of our methods enable comparison and meta-analysis of data generated from different platforms.

Another limitation was due to the same sample size in each dataset. Four datasets had less than 50 participants. Furthermore, the response rate was heavily tilted toward either side in several studies with either a very high or very low response rate. Therefore, meta-analysis was only applied to those datasets having a response rate between 10–90%.

## 5. Conclusions

A new and simple to analyze peripheral blood biomarker is introduced here which can be readily incorporated into the routine clinical laboratory. It could predict seroconversion status early after influenza vaccination. A global-wide vaccination campaign is underway to control the COVID-19 pandemic. Due to using the same mechanism of antibody production, Direct B lymphocyte LS-TA will also be a useful companion assay for the practice of personalized vaccination.

## Figures and Tables

**Table 1:** List of PBMC gene expression datasets used in this study. (n.a.: not available)

Data series Accession Number	Type of Blood Samples (WB or PBMC)	Total No. of Samples (All Time Points)	No. of QC Failed Samples	No.of Non-Responders (NR)	No.of Responders (R)	Sex (% Female)	% Young (<65 Year-Old)	References
GSE29614	PBMC	18	-	2	7	59%	100%	[7]
GSE29615	PBMC	55	-	26	1	55%	100%	[7]
GSE29617	PBMC	53	2	7	19	67%	100%	[7]
GSE101709	PBMC	41	4	11	8	64%	48%	[17]
GSE101710	PBMC	31	-	5	10	49%	50%	[17]
GSE59635	PBMC	36	1	7	10	67%	58%	[18]
GSE59654	PBMC	76	3	24	14	58%	41%	[18]
GSE59743	PBMC	50	-	9	11	60%	50%	[18]
GSE45764	Paired PBMC and Purified B lymphocytes	104	6			n.a.		[6]

**Table 2:** Shortlisted potential B lymphocyte informative genes in RNAseq dataset GSE45764. Cell-type informative reference genes could be selected among those with low CV%. Two genes stand out among peers of other well-recognized B cell markers: TNFRSF13C and FCRLA, which were further analyzed as B cell informative reference genes for this manuscript. (Both TNFRSF13C and FCRLA used as reference gene in the meta-analyses of Direct LS-TA biomarkers are shown in bold font).

Gene Symbol	Gene Expression Fold Difference between Purified B lymphocytes and PBMC Samples (X, Folds)	CV% in Purified B Lymphocyte Samples
*HLA-DOB*	13	11%
*CD19*	15	15%
*CD79B*	11	15%
*CD79A*	16	15%
*BANK1*	16	16%
*TNFRSF13C*	14	16%
*MS4A1 (CD20)*	16	17%
*BLK*	16	18%
*CD22*	16	18%
*FCRLA*	15	20%
*FCER2*	14	24%
*TNFRSF13B*	17	26%
*TCL1A*	14	30%
*TNFRSF17*	13	62%
*JCHAIN*	11	70%
*TXNDC5*	11	103%

**Table 3:** A shortlist of B-lymphocyte target genes that can be reliably measured in PBMC without the need for prior cell isolation.

B lymphocyte Informative Target Gene	B Lymphocyte Informative Reference Gene	Pearson’s Correlation Coefficient between Direct LSTA Assay in PBMC and Isolated B Cell Target Gene Expression
*IGHG1*	*TNFRSF13C*	0.96
*DSP*	*TNFRSF13C*	0.94
*IGHG3*	*TNFRSF13C*	0.94
*MZB1*	*TNFRSF13C*	0.93
*IGHA1*	*TNFRSF13C*	0.93
*IGLL5*	*TNFRSF13C*	0.93
*JCHAIN*	*TNFRSF13C*	0.92
*FCRL5*	*TNFRSF13C*	0.91
*IGHA2*	*TNFRSF13C*	0.9
*IGHM*	*TNFRSF13C*	0.9
*TNFRSF17*	*TNFRSF13C*	0.9
*TXNDC5*	*TNFRSF13C*	0.88
*IGKC*	*TNFRSF13C*	0.86
*COCH*	*TNFRSF13C*	0.85

**Table 4:** Four Direct B lymphocyte LS-TA biomarkers used in meta-analyses.

Target Gene of B Lymphocyte	Direct B Lymphocyte LS-TA Biomarkers Using PBMC Data
TNFRSF17	TNFRSF17: TNFRSF13C (TNFRSF13C as ref. gene)
	TNFRSF17: FCRLA (FCRLA as ref. gene)
TXNDC5	TXNDC5: TNFRSF13C (TNFRSF13C as ref. gene)
	TXNDC5: FCRLA (FCRLA as ref. gene)

## Data Availability

Data are available from NCBI GEO.

## Supplementary Materials

The following are available online at https://www.mdpi.com/article/10.3390/genes12070971/s1, Figure S1: Shows the relationship between required fold difference in TA between purified cell-type samples and cell mixtures in two scenarios, (1) B cells in WB (given proportional cell count as 5%) and (2) B cells in PBMC (given proportion cell count as 10%). The required TA fold difference for subpopulation informative genes are 10× and 5× for (1) and (2), respectively. Figure S2: Example of outliner samples in GSE59654, which are labeled as outline by Mahalanobis distance metrics based on (A) a list of housekeeping genes and (B) a list of leukocyte cell-type-specific genes. The three outliner samples called in both (A) and (B) are shown as red symbols on (C) scatter plots of housekeeping genes. Figure S3: Significant correlations were confirmed between Direct LS-TA of TNFRSF17 measured in PBMC samples (*Y*-axis, a ratio of TNFRSF17 and TNFRSF13C) and TA of purified B lymphocytes (ground-truth, *X*-axis) in multiple datasets. Supplementary Document: workflow flowchart.
