# Supplementary figures and images for: Direct Measurement of B Lymphocyte Gene Expression Biomarkers in Peripheral Blood Transcriptomics Enables Early Prediction of Vaccine Seroconversion
Source: Genes (Basel). 2021 Jun 25;12(7):971. doi: 10.3390/genes12070971 (PMC8304400; doi:10.3390/genes12070971)

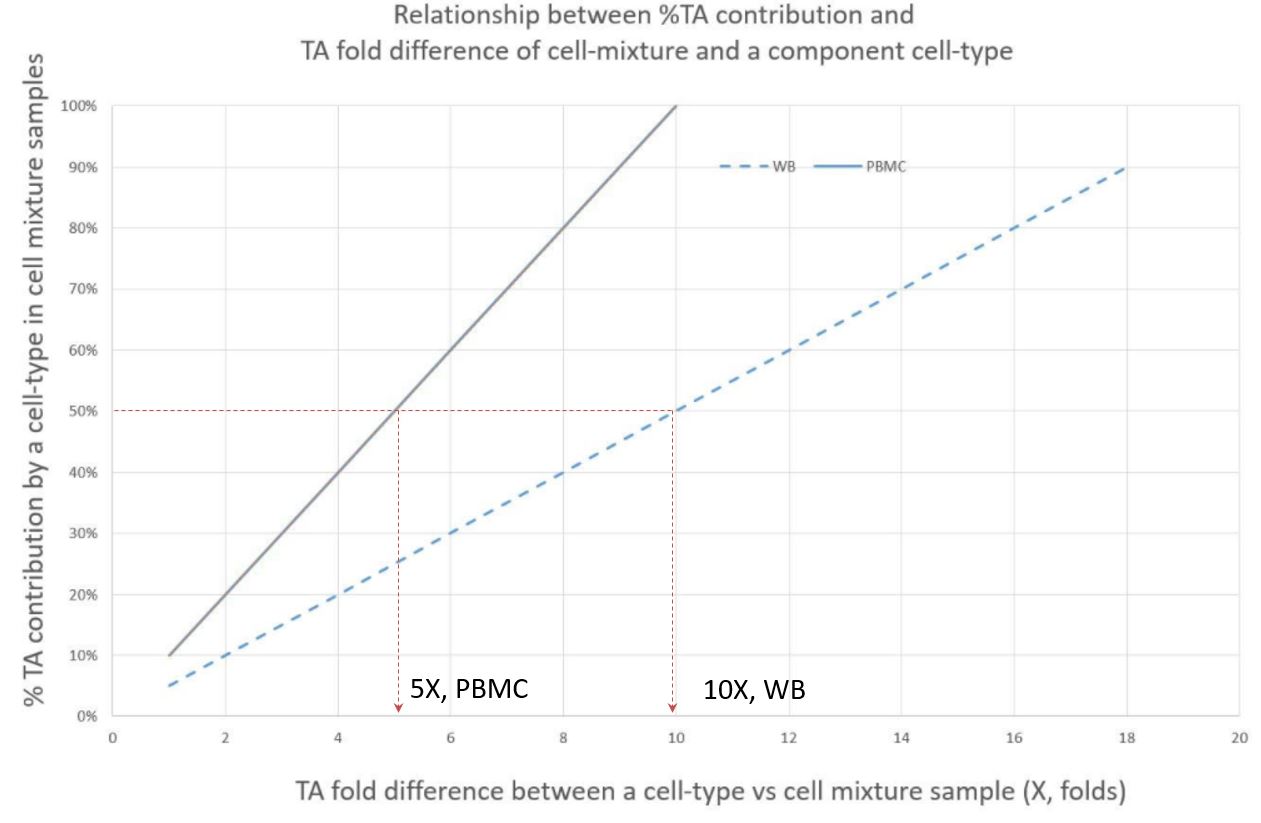

Supplement: Supplementary file 1 [file genes-12-00971-s001.zip › suppl figure 1.JPG]

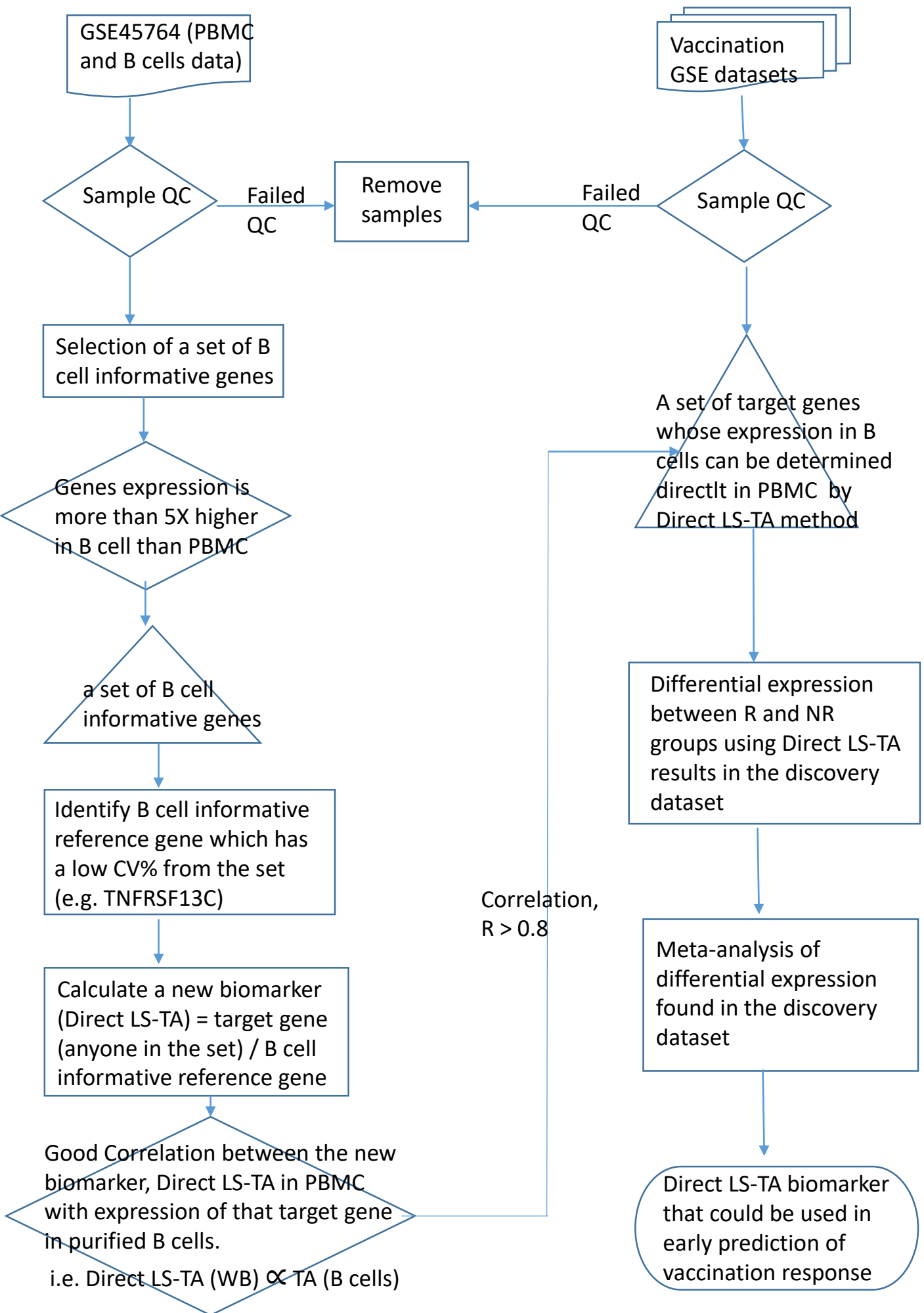

Supplement: Supplementary file 1 [file genes-12-00971-s001.zip › supplementary workflow.pdf]

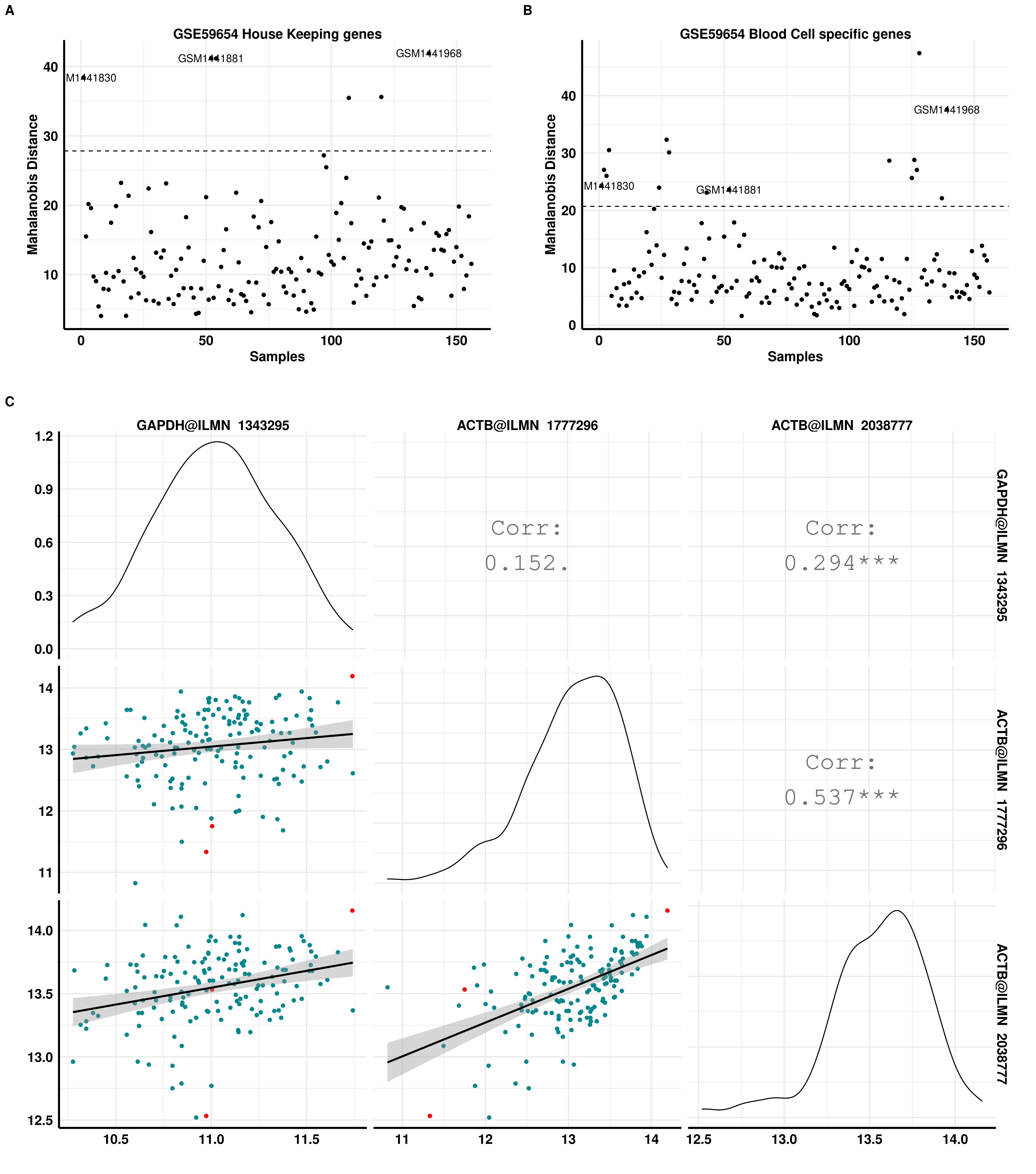

Supplement: Supplementary file 1 [file genes-12-00971-s001.zip › Supplymentary_Figure2.tiff]

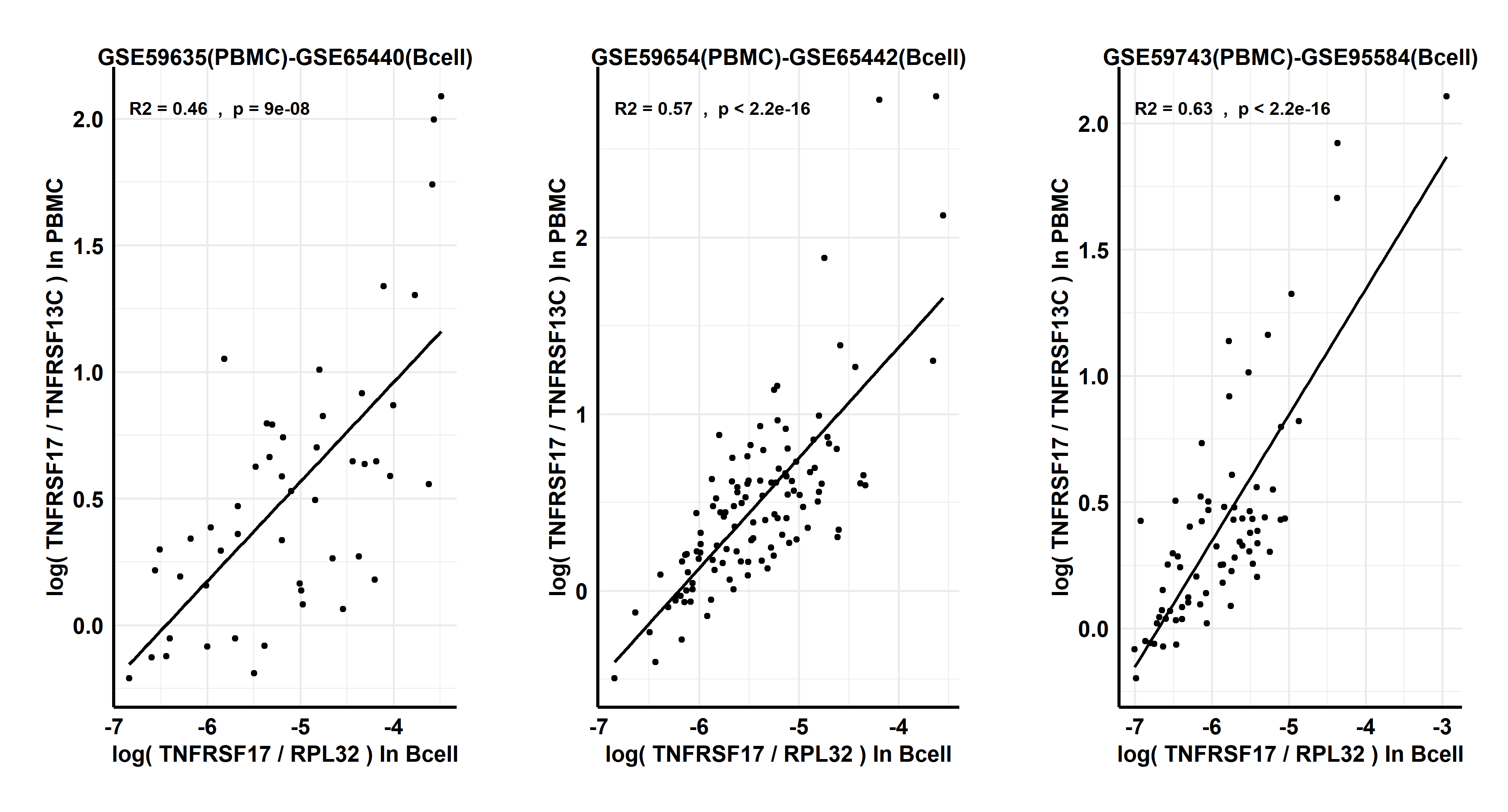

Supplement: Supplementary file 1 [file genes-12-00971-s001.zip › supplymentary_figure3.tiff]
